# Effects of different contact angles during forefoot running on the stresses of the foot bones: a finite element simulation study

**DOI:** 10.3389/fbioe.2024.1337540

**Published:** 2024-02-08

**Authors:** Huiyu Zhou, Datao Xu, Wenjing Quan, Ukadike Chris Ugbolue, Yaodong Gu

**Affiliations:** ^1^ Faculty of Sports Science, Ningbo University, Ningbo, China; ^2^ School of Health and Life Sciences, University of the West of Scotland, Paisley, United Kingdom; ^3^ Faculty of Engineering, University of Pannonia, Veszprem, Hungary

**Keywords:** contact angle, forefoot running, finite elements, foot, foot injury

## Abstract

**Introduction:** The purpose of this study was to compare the changes in foot at different sole-ground contact angles during forefoot running. This study tried to help forefoot runners better control and improve their technical movements by comparing different sole-ground contact angles.

**Methods:** A male participant of Chinese ethnicity was enlisted for the present study, with a recorded age of 25 years, a height of 183 cm, and a body weight of 80 kg. This study focused on forefoot strike patterns through FE analysis.

**Results:** It can be seen that the peak von Mises stress of M1-5 (Metatarsal) of ***a*** (Contact angle: 9.54) is greater than that of ***b*** (Contact angle: 7.58) and ***c*** (Contact angle: 5.62) in the three cases. On the contrary, the peak von Mises stress of MC (Medial Cuneiform), IC (Intermediate Cuneiform), LC (Lateral Cuneiform), C (Cuboid), N (Navicular), T (Tarsal) in three different cases is opposite, and the peak von Mises stress of c is greater than that of a and b. The peak von Mises stress of b is between a and c.

**Conclusion:** This study found that a reduced sole-ground contact angle may reduce metatarsal stress fractures. Further, a small sole-ground contact angle may not increase ankle joint injury risk during forefoot running. Hence, given the specialized nature of the running shoes designed for forefoot runners, it is plausible that this study may offer novel insights to guide their athletic pursuits.

## 1 Introduction

The sport of running has gained widespread popularity owing to its ease and accessibility, resulting in a steady rise in the number of individuals engaging in this physical activity with each passing year (Van Middelkoop et al., 2008). According to reports, running has been found to be an effective means of managing body weight, enhancing exercise tolerance, and mitigating the likelihood of cardiovascular disease (Taunton et al., 2002). Due to the fact that running does not necessitate specific facilities or gear, a considerable number of individuals opt to engage in running as a form of physical activity. The likelihood of sustaining an injury escalates with the increasing number of individuals engaging in running activities. Consequently, based on the sustained investigation and findings of researchers in recent times, it can be concluded that the impact load incurred during running strike patterns is significantly associated with the probability of sustaining injuries in the lower limbs (Lieberman et al., 2010; Thompson et al., 2015; Dempster et al., 2021; Pan et al., 2023; Yang et al., 2023).

A cohort of long-distance runners was subjected to analysis by researchers (Hasegawa et al., 2007; Larson et al., 2011; de Almeida et al., 2015), revealing that a significant majority of runners, approximately 95%, demonstrate a rearfoot strike pattern, whereby they make initial contact with the ground using their heel. The remaining proportion of individuals exhibit midfoot strike, flat foot landing, or forefoot strike, characterized by landing on the anterior part of the foot (Zhou and Ugbolue, 2019). However, there is a lack of scientific proof to establish which is the better running strike pattern. The investigation conducted by the researchers revealed that the long-distance runners who belong to the elite the level exhibited a forefoot and midfoot strike patterns during their running (Hanley et al., 2021). This finding suggests that rearfoot strike pattern may not be a favorable choice for high-performing athletes. Conversely, numerous sports companies are presently manufacturing forefoot running footwear tailored towards top-tier athletes, indicating a proclivity among elite long-distance runners to utilize forefoot running techniques.

The proposition put forth by the researchers suggests that forefoot strikes have the capacity to store a greater amount of elastic potential energy (Perl et al., 2012) and also have the ability to decrease vertical loading rates when compared to rearfoot strikes (Squadrone and Gallozzi, 2009; Crowell and Davis, 2011). On the other hand, certain academics posit that utilizing a forefoot strike while running can significantly impact the stresses on the foot bones, potentially resulting in metatarsal stress fractures (Li et al., 2017). At this point, we ask whether it is possible to change the contact angle between different sole-ground to further improve the lack of forefoot running? To the best of our knowledge, no studies have investigated the biomechanics of sole-ground contact angles. We speculate that this may be because the running characteristics of each athlete are different, and the running characteristics of different contact angles cannot be used as a classification index.

In situations where it is not feasible to meet the experimental requirements, the finite element analysis assumes a crucial role. The finite element method has the capability to accurately simulate real scenarios and provide insights into issues that are beyond the traditional biomechanics (Chang et al., 2008; Mabrouk et al., 2022). Finite element analysis is a reliable and controlled method for conducting foot simulations. This approach offers greater precision in defining individual modules, resulting in a more accurate representation of real situation (Cheung et al., 2009; Telfer et al., 2014; Wang et al., 2015; Wang et al., 2016). Moreover, finite element analysis finds application in various other analyses, including but not limited to car crash (Chang et al., 2008; Shin et al., 2012), running stance phase (Qian et al., 2013; Chen and Lee, 2015), and landing impact (Cho et al., 2009; Farhang et al., 2016).

The purpose of this study was to compare the changes in foot at different sole-ground contact angles during forefoot running. In light of the fact that elite long-distance runners predominantly utilize forefoot running techniques, an investigation to help forefoot runners better control and improve their technical movements by comparing different sole-ground contact angles is paramount. In addition, we further hope to provide inspiration for the future direction of running shoe design through this research. We hypothesized that the overall bone stress changes as the sole-ground contact angle changes. More specifically, perhaps the smaller the sole-ground contact angle, the greater the bone stress in the rear foot.

## 2 Methods

### 2.1 Participant

A male participant of Chinese ethnicity was enlisted for the present study, with a recorded age of 25 years, a height of 183 cm, and a body weight of 80 kg. This participant maintained a running habit for a long time, at least 3 times a week. The investigation focused on forefoot strike patterns. The subject’s lower limbs were devoid of any documented medical conditions, and no surgical interventions were detected within the participant’s medical history in the 12 months preceding the experiment that could have potentially influenced the results. Upon receiving a comprehensive explanation of the research’s purpose and methodology, the participant provided written consent in acknowledgement of their informed decision to participate. Approval for this study was obtained from the Ethics Committee of Ningbo University (protocol code: RAGH 20220918).

### 2.2 Biomechanics parameters collection and processing

**FIGURE 1:**
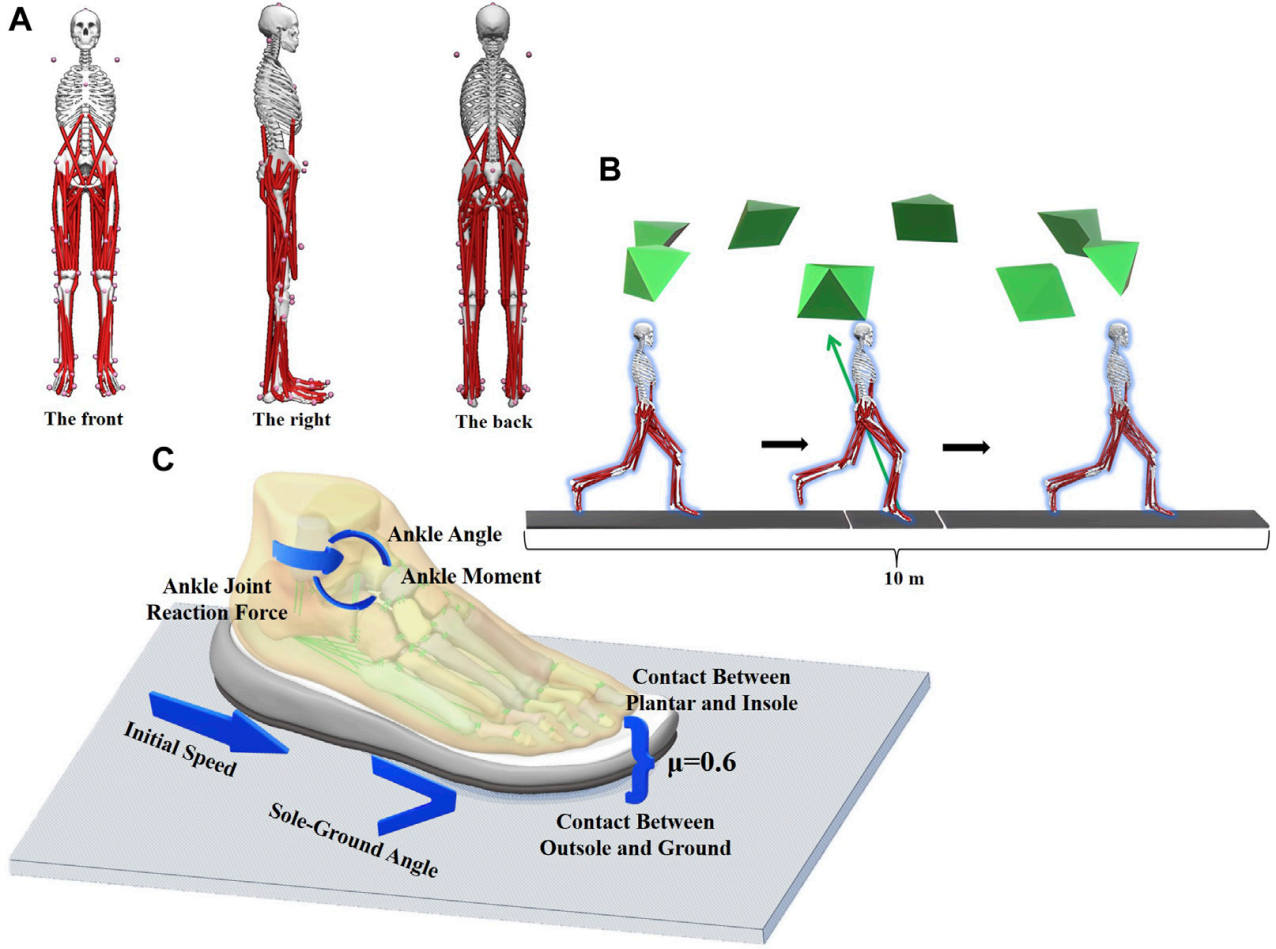
Acquisition of kinematic and dynamic data and setup of finite element boundary conditions are detailed.

All tests were conducted in a biomechanics laboratory, specifically the Research Academy of Grand Health at Ningbo University. The study employed a Kistler force platform and an eight-camera Vicon motion capture system (Oxford Metrics Ltd., Oxford, UK) to collect data on dynamics and kinematics, synchronized. The present investigation involved the acquisition of kinematic and dynamic data, which were respectively sampled at frequencies of 200 and 1000 Hz. Figure 1A displays the spatial distribution of 39 markers. The subject proceeded and ran on a 10-m running way at a speed of 3.3 m/s in order to collect kinetic information (Figure 1B). The infrared timers were placed on either side of the 10-m track to measure the participants’ running speed. The initial contact was operationally defined as the time when the ground reaction force (GRF) surpassed the 10 N threshold (Xu et al., 2022; Zhou et al., 2022). The subject conducted one hundred data trials.

**FIGURE 2:**
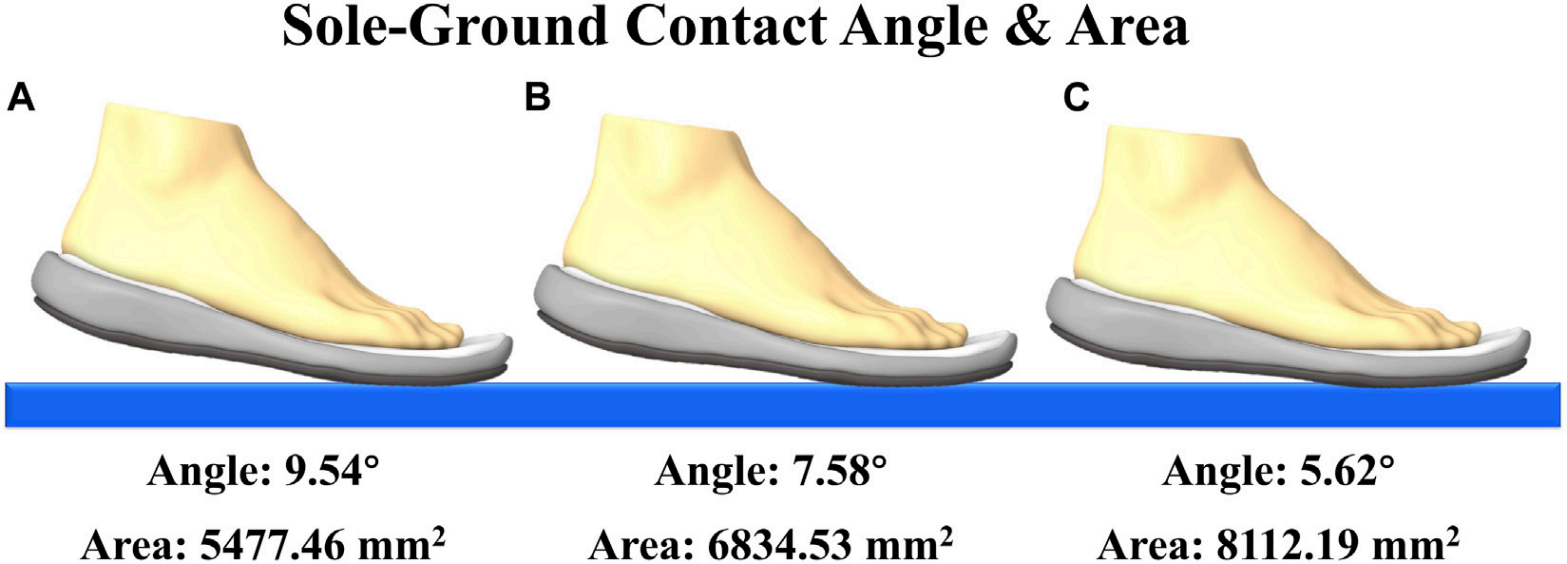
Schematic diagram of three different bottoming angles and the contact area between the shoe and the ground under different sole-ground contact angles.

The present study utilized OpenSim software (Stanford University in Stanford, CA, USA) to investigate and compute biomechanical parameters to take into the FE analysis. Three models were established in this study, each representing a distinct situation (Figure 2). Initially, the mean value of the sole-ground angle was computed for a sample of 100 data sets. This resulted in the establishment of the first model, with an angle of ***b*** (b = 7.58°) as the designated value. Moreover, we extracted the minimum angle ***c*** (c = 5.62°) and maximum angle ***a*** (a = 9.54°) from one hundred datasets to take into consideration for two additional situations of the angle between the sole and the ground.

### 2.3 The process of obtaining and reconstructing geometric data

The right foot of the participant was imaged using CT and MRI techniques with a 2 mm interval. The segmentation of the two-dimensional image was performed using Mimics 21.0 (Materialise, Leuven, Belgium), while the creation and refinement of the bone, ligaments, Achilles tendon, bulk soft tissue, and shoes were carried out using Geomagic Studio 2021 (Geomagic, Inc., Research Triangle Park, NC, United States). The SolidWorks 2017 software was utilized for importing the components and subsequently converting them into solids (SolidWorks Corporation, Waltham, MA, United States). The structure of cartilage has been simulated through the construction of a solid material that fills the space between the surfaces of two adjacent bones.

The contacts of the models were meshed and established utilizing Workbench 2021 (ANSYS, Inc. located in Canonsburg, PA, USA). Tetrahedral meshes were employed to decompose each solid. The age matching model, having undergone successful mesh convergence testing, underwent modifications to the mesh sizes of the bulk soft tissue, bone, shoes, and cartilage at 3 mm, 2 mm, 2 mm, and 0.5 mm, respectively. Furthermore, the process of local refining was executed with consideration given to the geometric characteristics of the contact zone. The Workbench software facilitated the automated detection of component contacts. Possible contact pairings were generated through the utilization of an algorithm that relies on surface proximity. The physical interaction between the bone surface and cartilage was emulated through direct face-to-face contact. The surface of the bone made frictionless contact with the cartilage. The soft tissue that was encapsulated served as an anchor for both the bones and cartilage. A contact surface with a friction coefficient of 0.6 was employed to replicate the interaction among the foot, shoes, and ground. All the constituents of the footwear were assembled to bond, along with the other remaining elements.

### 2.4 Boundary and loading condition

An explicit dynamic solver was used to perform a simulation of the forefoot running stance phase. First, fix the ground, and set and define the position of the foot model. In the finite element model, the ankle joint angle was set by adjusting the angle between the tibial axis and the longitudinal axis of the foot on the sagittal plane. The global coordinate system remained consistent with the original coordinate system of OpenSim (Delp et al., 2007). The set initial velocity was added to the finite element model. The slipring connectors and tibiotalar articular surface of the talus were applied to the ankle joint moment and ankle joint reaction force, respectively. The joint force of the MPJ was exerted onto the upper surface of the middle cuneiform bone to simulate the force of inertia experienced during landing. Calculated by OpenSim, the time from initial contact to maximum ground reaction force was 0.115 s, so the time step was set to 0.115 s. Table 1 displays the specific value of the loading condition.

**TABLE 1:** Running kinematics and kinetics gait characteristics.

Experimental variables	
Initial Speed	3.3 m/s
Ankle Angle	7.37° Plantarflexion
Ankle Moment	3.56 Nm/kg
Ankle Joint Force	18.33 N/kg
MPJ Force	9.77 N/kg
Peak GRF	2.39 BW
Contact Time	0.115 s

Note: BW = body weight.

All materials, except for the encapsulated soft tissue and skin, were assumed to be isotropic and linear elastic materials, and their properties were obtained from prior research (Crowninshield and Nakamura, 1981; Siegler et al., 1988; Hurschler et al., 1994; Kitaoka et al., 1994; Cook and McDonagh, 1996; Davis et al., 1996; Milz et al., 1998; Gefen et al., 2000; Kura et al., 2001; Wren et al., 2001; Bayraktar et al., 2004; Cheung et al., 2005; Cheung and Zhang, 2005; Wu, 2007; Pailler-Mattei et al., 2008; Gu et al., 2010; Chen et al., 2012; Wong et al., 2016; Wang et al., 2021). The two material parameters, including Young’s modulus (E) and Poisson’s ratio (v), were chosen to characterize the elastic properties of the material. Table 2 enumerates the material properties of each constituent.

**TABLE 2:** Material properties of the components in the finite element model.

		Material property	Young’s modulus (MPa)	Poisson’s ratio	Density (kg/m3)
Skin	Tetrahedral solid	Hyperelastic (first-order Ogden model, μ=0.122 kPa,α=18 )	—	—	950
Bulk Soft Tissue	Tetrahedral solid	Hyperelastic (second-order polynomial strain, $C_{10}=0.8556,C_{01}=0.05841,C_{20}=0.03900,C_{11}=0.02319,C_{02}=0.00851,D_{1}=3.65273$ )	—	—	950
Bone	Tetrahedral solid	Linearly Elastic	7300	0.3	1500
Cartilage	Tetrahedral solid	Linearly Elastic	1	0.4	1050
Ligaments	Two-node truss	Linearly Elastic	260	0.4	1000
Profundal Fascia	Two-node truss	Linearly Elastic	190	0.4	950
Plantar Fascia	Two-node truss	Linearly Elastic	350	0.4	1000
In-Sole	Tetrahedral solid	Linearly Elastic	1.98	0.35	2300
Mid-Sole	Tetrahedral solid	Linearly Elastic	2.49	0.35	2300
Out-Sole	Tetrahedral solid	Linearly Elastic	3.85	0.4	2300
Plate	Tetrahedral/Hexahedral solid	Linearly Elastic	17000	0.4	1000

## 3 Results

**FIGURE 3:**
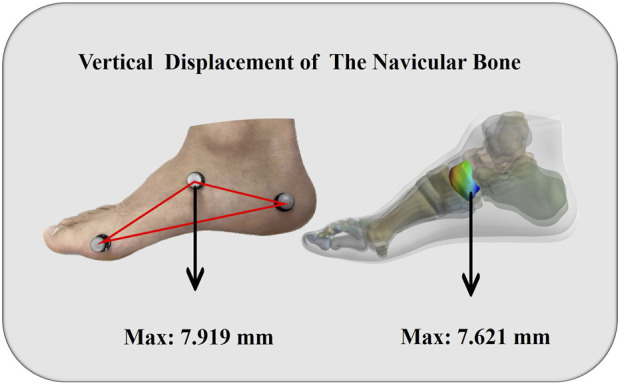
Vertical displacement validation of model.

To verify the accuracy of the FE foot model, a simulation of forefoot running was conducted and subsequently compared to the navicular bone’s deformation. The displacement of the navicular bone is utilized as a surrogate measure for the foot deformation index in clinical contexts. The tuberosity of the navicular bone on the medial side is frequently employed as the reference point in manual measurements. Graphing the vertical displacement from a given node during the period in which the entire body weight is being sustained. Using 14 data sets, the intraclass correlation coefficient (ICC) were used to assess the level of agreement between *in-vivo* measurements and predictions. According to (Koo and Li, 2016), the ICC estimate was classified as weak below 0.50, moderate between 0.50 and 0.75, strong between 0.75 and 0.9, and excellent reliability beyond 0.90. Our results show that the ICC test displayed an excellent ICC score (0.95). Figure 3 illustrates the comparison between the measured deformation of the navicular bone (Picciano et al., 1993; Nielsen et al., 2009) and the result obtained through finite element simulation.

**FIGURE 4:**
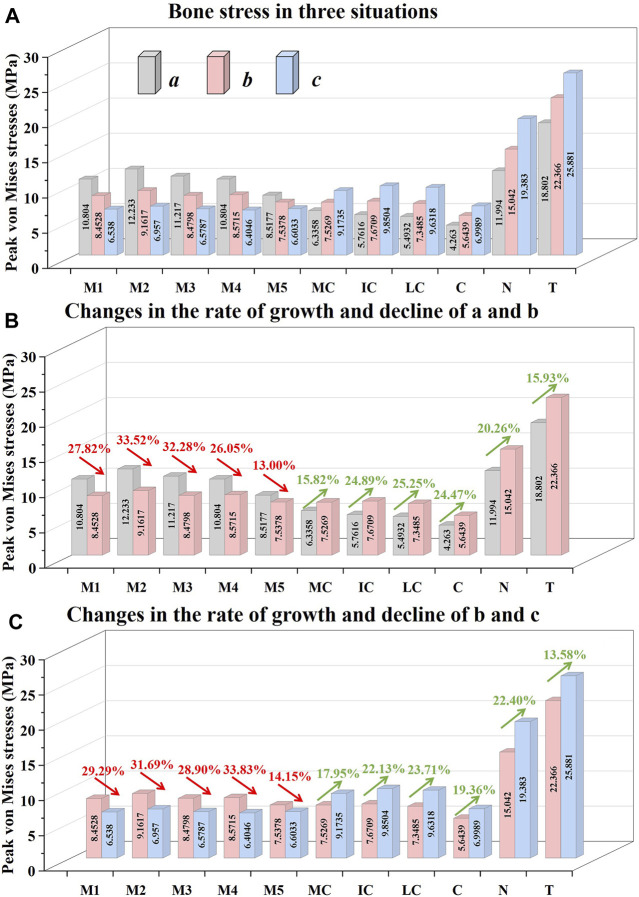
Illustration of the peak von Mises stress values and percentage increases and decreases of the different bones in three different situations.

**FIGURE 5:**
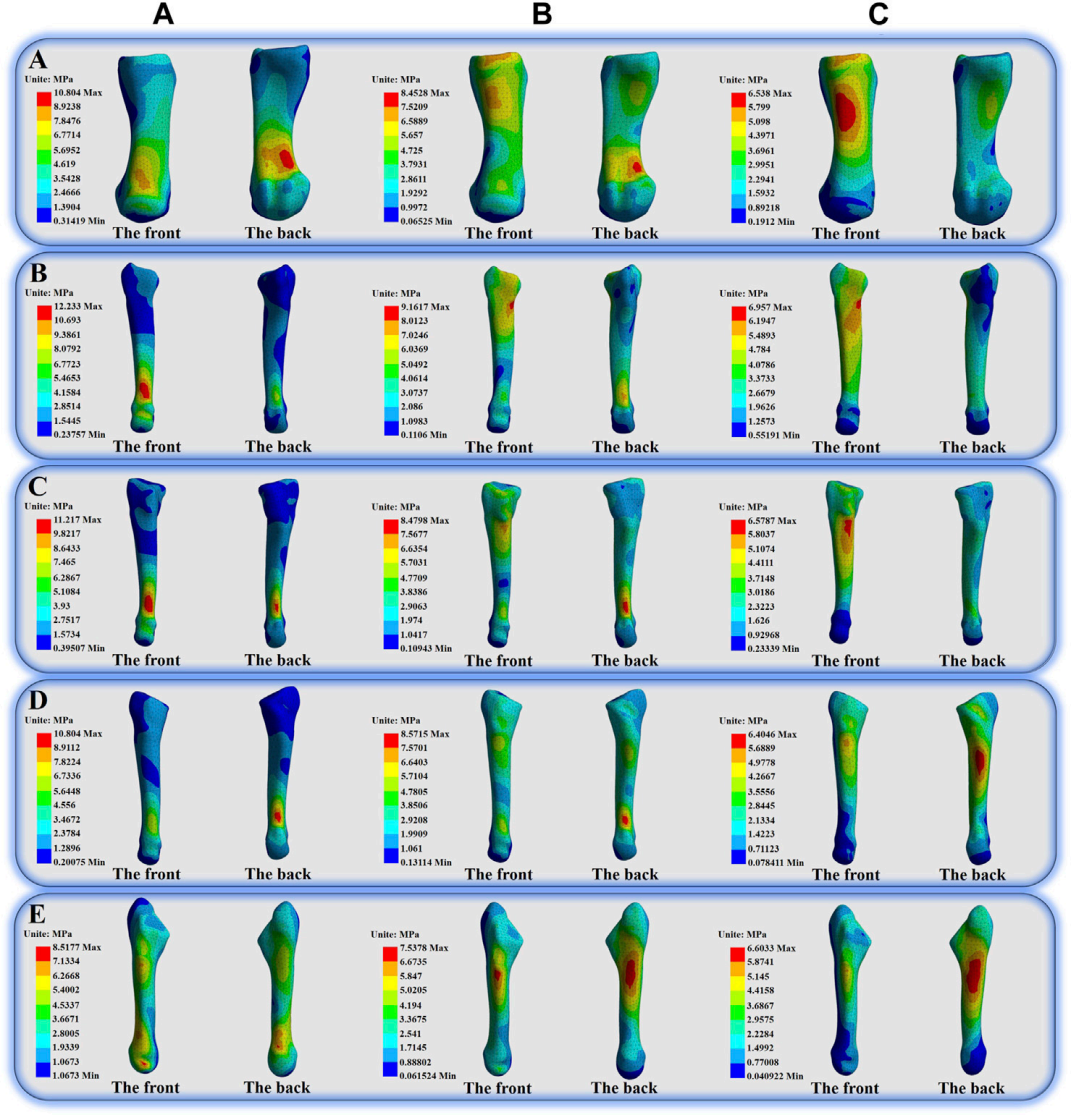
Illustration of the von Mises stress distribution of the first-fifth Metatarsal bones.

**FIGURE 6:**
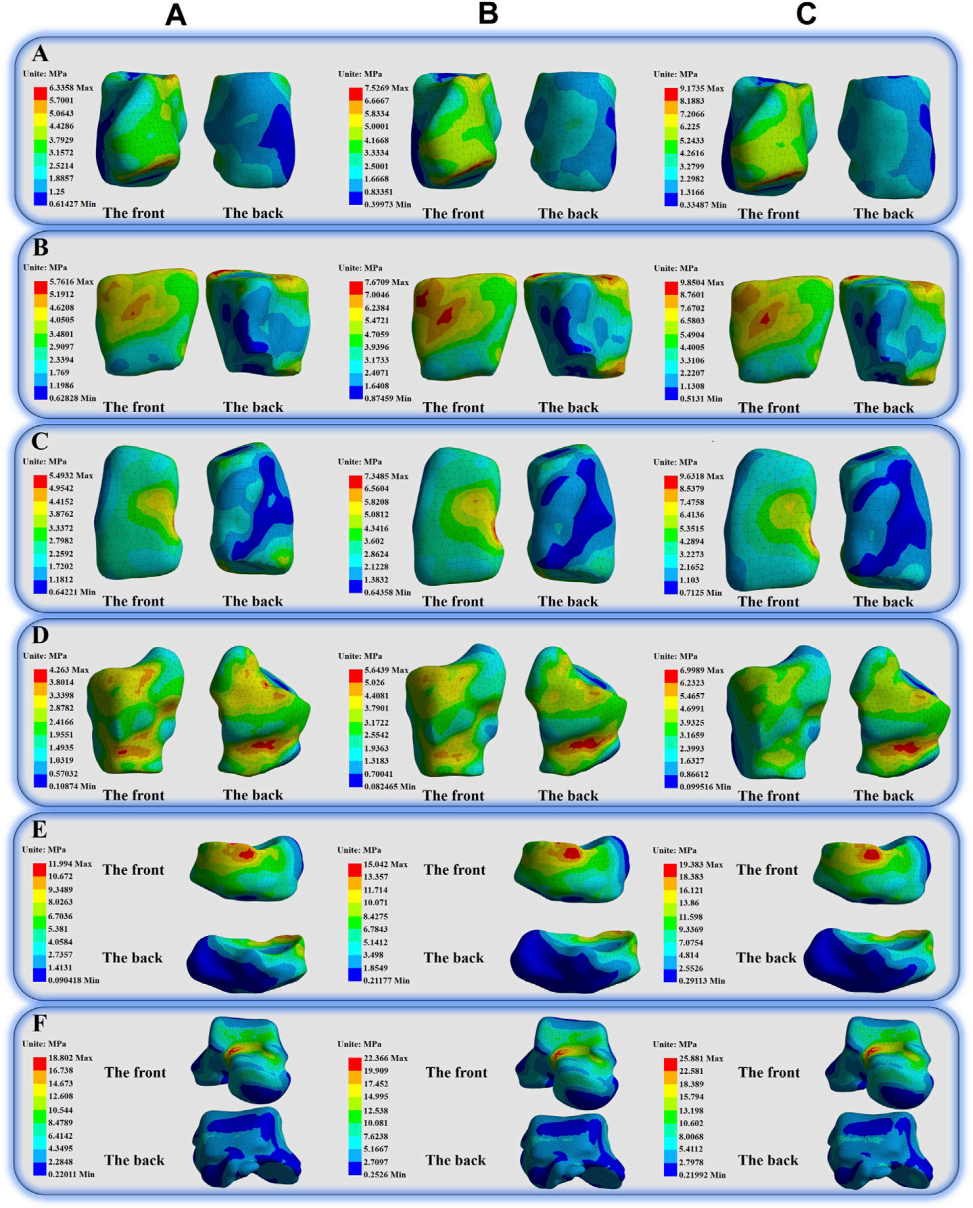
Illustration of the von Mises stress distribution of the Medial Cuneiform, Intermediate Cuneiform, Lateral Cuneiform, Cuboid, Navicular and Tarsal.

Figure 4A shows the peak von Mises stress of the foot bones in three different cases. It can be seen that the peak von Mises stress of M1-M5 of a is greater than that of ***b*** and ***c*** in the three cases. On the contrary, the peak von Mises stress of MC, IC, LC, C, N, T in three different cases is opposite, and the peak von Mises stress of ***c*** is greater than that of ***a*** and ***b***. The peak von Mises stress of b is always between ***a*** and ***c***. The peak von Mises stress values of the three situations of M1 are ***a*** = 10.804 MPa, ***b*** = 8.4528 MPa, ***c*** = 6.538 MPa (Figure 5A); the peak von Mises stress values of the three situations of M2 are ***a*** = 12.233 MPa, ***b*** = 9.1617 MPa, ***c*** = 6.957 MPa (Figure 5B); the peak von Mises stress values of the three situations of M3 are ***a*** = 11.217 MPa, ***b*** = 8.4798 MPa, ***c*** = 6.5787 MPa (Figure 5C); the peak von Mises stress values of the three situations of M4 are ***a*** = 10.804 MPa, ***b*** = 8.5715 MPa, ***c*** = 6.4046 MPa (Figure 5D); the peak von Mises stress values of the three situations of M5 are ***a*** = 8.5177 MPa, ***b*** = 7.5378 MPa, ***c*** = 6.6033 MPa (Figure 5E); the peak von Mises stress values of the three situations of MC are ***a*** = 6.3358 MPa, ***b*** = 7.5269 MPa, ***c*** = 9.1735 MPa (Figure 6A); the peak von Mises stress values of the three situations of IC are ***a*** = 5.7616 MPa, ***b*** = 7.6709 MPa, ***c*** = 9.8504 MPa (Figure 6B); the peak von Mises stress values of the three situations of LC are ***a*** = 5.4932 MPa, ***b*** = 7.3485 MPa, ***c*** = 9.6318 MPa (Figure 6C); the peak von Mises stress values of the three situations of C are ***a*** = 4.263 MPa, ***b*** = 5.6439 MPa, ***c*** = 6.9989 MPa (Figure 6D); the peak von Mises stress values of the three situations of N are ***a*** = 11.994 MPa, ***b*** = 15.042 MPa, ***c*** = 19.383 MPa (Figure 6E); and the peak von Mises stress values of the three situations of T are ***a*** = 18.802 MPa, ***b*** = 22.366 MPa, ***c*** = 25.881 MPa (Figure 6F).

Figure 4B depicts the percentage variation in the peak von Mises stress values of a certain bone in situations ***a*** and ***b***. When comparing ***a*** and ***b***, the M1 of **a** decreased by 27.82%; the M2 of **a** decreased by 33.52%; the M3 of **a** decreased by 32.28%; the M4 of ***a*** decreased by 26.05%; the M5 of ***a*** decreased by 13.00%; the MC of ***a*** increased by 15.82%; the IC of ***a*** increased by 24.89%; the LC of ***a*** increased by 25.25%; the C of ***a*** increased by 24.47%; and the N of ***a*** increased by 20.26%; the T of ***a*** increased by 15.93%.

Figure 4C depicts the percentage variation in the peak von Mises stress values of a certain bone in situations ***b*** and ***c***. When comparing ***b*** and ***c***, the M1 of ***b*** decreased by 29.29%; the M2 of ***b*** decreased by 31.69%; the M3 of ***b*** decreased by 28.90%; the M4 of ***b*** decreased by 33.83%; the M5 of ***b*** decreased by 14.15%; the MC of ***b*** increased by 17.95%; the IC of ***b*** increased by 22.13%; the LC of ***b*** increased by 23.71%; the C of ***b*** increased by 19.36%; the N of ***b*** increased by 22.40%; and the T of ***b*** increased by 13.58%.

## 4 Discussion

The purpose of this study was to compare the changes in foot bone stress at different sole-ground contact angles during forefoot running. We tried to help forefoot runners better control and improve their technical movements by comparing different sole-ground contact angles. In addition, we further hope to provide inspiration for the future direction of running shoe design through this research. We hypothesized that the overall bone stress changes as the sole-ground contact angle changes. More specifically, the smaller the sole-ground contact angle, the greater the bone stress in the rear foot. The findings of this research align with our initial hypothesis.

Metatarsal stress fractures constitute approximately 10%–20% of all stress fractures observed in athletes and exhibit a notable prevalence among runners (Matheson et al., 1987). The possibility of experiencing metatarsal stress fractures is greater for forefoot runners as compared to rearfoot runners (Kernozek et al., 2014). Prior research has shown that assessing the magnitude of metatarsal stress values is a crucial factor in the assessment of metatarsal stress fractures (Madjarevic et al., 2009). It can be seen that from our findings reported in Figure 4A the peak von Mises stress change of the foot bones will change with respect to changes to the sole-ground contact angle. In the situation of ***a***, the peak von Mises stress of the five metatarsal bones is larger than that of the other two situations. This observation suggests that a decrease in sole-ground contact angle may be associated with a lower probability of metatarsal stress fractures. In comparison to previous studies (Matheson et al., 1987; Madjarevic et al., 2009; Kernozek et al., 2014), we observed that situation ***c*** exhibited the lowest probability of stress fractures occurring in the metatarsals.

On the contrary, the MC, IC, LC, C, N and T of ***a*** produced less peak von Mises stresses when compared to the other two situations. Reducing the sole-ground contact angle results in a decrease in the peak von Mises stress on the metatarsals; however, this concurrently leads to an increase in the peak von Mises stress exerted on the midfoot bones. Prior research has suggested that this particular condition may also result in an elevated potential to midfoot fractures (Zhang and Zhang, 2022). At the same time, the researchers further pointed out that the T bone exhibits frequent movement along the coronal axis within the sagittal plane, while infrequently undergoing non-physiological joint movements. Therefore, a situation like ***c*** may not place the talus in a particularly risky state. In other words, even though we still need more evidence to be certain, it seems that a minor sole-ground contact angle may not raise the risk of injury to the ankle joint. Also, it might be an effective way for reducing the peak von Mises stress on the metatarsals without causing further damage to the ankle joint.

A preliminary speculation of this outcome suggests that the midfoot bone serves as a central component of the skeletal structure situated between the forefoot and the rear foot. Its primary function is to facilitate the transfer of impact from the tibia, fibula, and heel bone to the five metatarsals, ultimately redistributing the load to the forefoot. The navicular and cuneiform bones located in the midfoot region play a crucial role in the mechanical transmission of the foot. The transmission of impact through the human foot occurs via the tibia and fibula, which subsequently transmit it through the talus to the navicular bone. At the navicular bone, the load is transmitted to the three cuneiform bones and ultimately to the metatarsals. In brief, the research findings presented in this study may be attributed to the principle of leverage. In the stance phase of running, the longitudinal foot is considered a lever with the ground contact part serving as the fulcrum. A decrease in sole-ground contact angle could be positively correlated with an increase in peak von Mises stress on the midfoot and rearfoot bones.

Eliud Kipchoge stands as the only athlete to have accomplished a marathon in a time frame of under 2 hours. Whilst acknowledging the existence of multiple contributing factors, it is noteworthy that the utilization of specific running shoes (namely, the Nike ZoomX Alphafly) played a significant role. Subsequently, forefoot running shoes garnered increased attention from researchers (Lu et al., 2022; Quan et al., 2023), prompting other sports brands to engage in the development and innovation of such footwear. Hence, given the specialized nature of the running shoes designed for forefoot runners, it is plausible that this study may offer novel insights to guide their athletic pursuits. Modifying the sole-ground contact angle has the potential of decreasing the possibility of injury to the athlete or enhance their athletic competence.

It is imperative that we acknowledge that the current study exhibits limitations. Initially, the selection process for this study involved the inclusion of a single male participant who exhibited good health. Due to inherent individual variability, the conclusions drawn from the study may vary. Secondly, the ligaments were assumed to possess linear elastic properties, despite the fact that they may exhibit hyperelastic or viscoelastic behavior. The chosen methodology may result in an underestimation of the collective rigidity of the model. However, this approach is frequently employed in finite element foot models as a means of achieving computational efficiency. Additionally, it is important to note that the material properties of certain foot ligaments are not completely represented (Morales-Orcajo et al., 2016). Furthermore, the boundary conditions for all three models, each characterized by distinct sole-ground contact angles, are identical. Ultimately, different material property, mesh size and mesh behavior et al. settings conditions will also have a great impact on the final result. It must be acknowledged that this situation is inconsistent with the actual state of situations. However, through the manipulation of variables, we can delve more profoundly into the potential underlying principles.

## 5 Conclusion

In summary, the present study was to investigate the changes in foot bone stress at different sole-ground contact angles during forefoot running. We found that a decrease in sole-ground contact angle may be associated with a lower probability of metatarsal stress fractures. We further found that a minor sole-ground contact angle may not raise the risk of injury to the ankle joint; and it might be an effective way for reducing the peak von Mises stress on the metatarsals without causing further damage to the ankle joint. Going forward further work would involve an investigation of the maximum principle stresses, directional stresses (i.e., what bones are in compression or in tension) and different failure theories. Fatigue failures in bone and a review of the stress fractures using the model would be worth investigating.

## Data Availability

The original contributions presented in the study are included in the article/Supplementary material, further inquiries can be directed to the corresponding author.
